# Extravillous trophoblasts invade more than uterine arteries: evidence for the invasion of uterine veins

**DOI:** 10.1007/s00418-016-1509-5

**Published:** 2016-10-24

**Authors:** Gerit Moser, Gregor Weiss, Monika Sundl, Martin Gauster, Monika Siwetz, Ingrid Lang-Olip, Berthold Huppertz

**Affiliations:** Institute of Cell Biology, Histology and Embryology, Medical University of Graz, Harrachgasse 21/7, 8010 Graz, Austria

**Keywords:** Extravillous trophoblasts, Endovascular trophoblasts, Endoglandular trophoblasts, Uterine veins, Invasion, Placenta

## Abstract

During the first trimester of pregnancy, extravillous trophoblasts (EVTs) invade into the decidual interstitium to the first third of the myometrium, thereby anchoring the placenta to the uterus. They also follow the endovascular and endoglandular route of invasion; plug, line and remodel spiral arteries, thus being responsible for the establishment of hemotrophic nutrition with the beginning of the second trimester and invade and open uterine glands toward the intervillous space for a histiotrophic nutrition during the first trimester. The aim of this study was to provide proof that uterine veins are invaded by EVTs similar to uterine arteries and glands in first trimester of pregnancy. Therefore, serial sections from in situ first trimester placenta were immuno-single- and immuno-double-stained to distinguish in a first step between arteries and veins and secondly between invaded and non-invaded vessels. Subsequently, invasion of EVTs into uterine vessels was quantified. Our data show that uterine veins are significantly more invaded by EVTs than uterine arteries (29.2 ± 15.7 %) during early pregnancy. Counted vessel cross sections revealed significantly higher EVT invasion into veins (59.5 ± 7.9 %) compared to arteries (29.2 ± 15.7 %). In the lumen of veins, single EVTs were repeatedly found, beside detached glandular epithelial cells or syncytial fragments. This study allows the expansion of our hitherto postulated concept of EVT invasion during first trimester of pregnancy. We suggest that invasion of EVTs into uterine veins is responsible the draining of waste and blood plasma from the intervillous space during the first trimester of pregnancy.

## Introduction

Extravillous trophoblasts (EVTs) of the decidua basalis originate from trophoblastic cell columns of anchoring villi and invade into maternal uterine decidua finally reaching the inner third of the myometrium (Kaufmann et al. 2003). Invasion of EVTs serves to attach the placenta to the uterus (interstitial invasion) and is responsible for the accession of nutrients to the embryo within the placenta (endovascular and endoglandular invasion) (Moser et al. 2010, 2015; Pijnenborg et al. 2006, 2011).

Spontaneous decidualization is initiated in the late secretory phase (around day 22 of the menstrual cycle). Thus, decidualization typically starts prior to implantation under the influence of progesterone, and the first signs become visible on day 23 of the menstrual cycle. Following implantation, decidualization is enhanced by high levels of progesterone (de Ziegler et al. 1998). It involves the endometrial stroma and the vessel walls of the spiral arteries. Thereafter, the presence of endovascular trophoblasts is responsible for the onset of “trophoblast-associated remodeling” of the spiral arteries. During this well-described process, an amorphous fibrinoid material is deposited and replaces the original smooth muscle layer of the vasculature, together with a complete loss of the elastic lamina (Pijnenborg et al. 2011). The decidua-associated spiral artery remodeling shows swelling and vacuolation of the endothelium together with disintegration of the vascular smooth muscle layer and swelling of individual muscle cells. Uterine natural killer cells and macrophages are important triggers of this early remodeling step (Pijnenborg et al. 2011).

The structure of uterine veins differs from that of spiral arteries in some minor aspects. The muscular layer is reduced, and near the venous openings to the intervillous space, smooth muscle cells are usually completely absent. In the rhesus monkey, the endothelium in venous segments near the intervillous space has sometimes been replaced by intraluminal trophoblast cells (Blankenship et al. 1993; Frank and Kaufmann 2006). However, so far nobody has had a specific look at uterine veins in the invaded regions of the first trimester decidua basalis. Aside that, in strongly invaded regions some of the arteries are not invaded at all, while veins seemed to be invaded by extravillous trophoblasts. Thus, the aim of this study was to provide proof that uterine veins are invaded by extravillous trophoblasts in the same manner as uterine arteries and glands in first trimester of pregnancy. Therefore, serial sections from in situ first trimester placenta were immuno-single- and immuno-double-stained to distinguish in a first step between arteries and veins and secondly between invaded and non-invaded vessels. Subsequently, invasion of EVTs into uterine vessels was quantified.

## Materials and methods

### Tissue collection

First trimester placentas were obtained from elective surgical terminations of pregnancies [gestational age (GA) 5–11 weeks, *n* = 41]. Informed consent was obtained from each woman included in the study with approval of the ethics committee of the Medical University of Graz. From every placenta, various tissue samples (villi, decidua basalis, decidua parietalis) were collected, depending on availability in the respective specimen. Invaded decidua basalis was identified in 16 (*n* = 16, GA 6–11 weeks) out of the 41 cases. For the preparation of formalin-fixed paraffin-embedded (FFPE) sections, tissues were fixed in 4 % neutrally buffered formalin for at least 24 h and routinely embedded in paraffin.

### Preparation of sections

Serial 5-µm paraffin sections were cut and placed on Superfrost Plus slides (Menzel, Braunschweig, Germany). FFPE sections were deparaffinized in xylene and rehydrated through a graded series of alcohol. Heat-induced antigen retrieval was performed in antigen retrieval solution at pH 9 (Leica Biosystems, Nussloch, Germany) in a pressure cooker (Model DC2002, Biocare Medical, Concord, USA) for 7 min at 120 °C before immunohistochemistry.

### Immunohistochemistry

Immunohistochemistry was performed using the Ultravision LP detection system (Thermo Scientific, Fremont, USA) according to the manufacturer’s instructions. Primary antibodies were diluted in antibody diluent (Dako, Carpinteria, USA) and applied for 30 min at room temperature to the tissue sections. Table 1 lists details of all antibodies used and their respective dilutions. Sections were counterstained with Mayer’s hemalaun and mounted with Kaiser’s glycerol gelatin (Merck, Vienna Austria). Negative controls were incubated with the appropriate IgG fractions as isotype controls (Table 1).

**Table 1:** Antibodies

Antigen/antibody (clone/cat no)	Company	Concentration (stock solution)	Dilution IHC	Host/isotype
KRT7 (APO6204PU-N)	Acris (Herford, Germany)	1 mg/ml	1:1000	Rabbit IgG pc
Human desmin (M 0760)	DakoCytomation (Pleasanton, Canada)	111 mg/L	1:100	Mouse IgG mc
HLA-G (4H84/557577)	BD Pharmingen (Vienna, Austria)	0.5 mg/ml	1:1000	Mouse IgG mc
VWF (F3520)	Sigma-Aldrich (St. Louis, USA)	7.1 mg/ml	1:1000	Rabbit IgG pc
Smooth muscle actin (1A4/M0851)	DakoCytomation (Pleasanton, Canada)	71 mg/L	1:1000	Mouse IgG mc
EphB4 (D1C7 N)	Cell signaling (Danvers, USA)	0.095 mg/ml	1:50	Rabbit IgG mc
Mouse IgG1 (DAK-GO1)	Dako (Carpinteria, USA)	100 mg/l	Matched to each primary antibody	Mouse IgG mc
Rabbit immunoglobulin fraction (X 0903)	Dako (Carpinteria, USA)	20 mg/ml	Matched to each primary antibody	Rabbit IgG pc

*mc* monoclonal, *pc* polyclonal, *IHC* immunohistochemistry

### Immunohistochemical double staining

Immunohistochemical double staining was performed using the Multivision Polymer Detection system (MultiVision anti-rabbit/AP + anti-mouse/HRP polymers; Thermo scientific, Fremont, USA) according to the manufacturer’s instructions. Primary antibodies were diluted and mixed in antibody diluent (Dako, Carpinteria, USA), and this primary antibody cocktail was applied for 30 min at room temperature to the tissue sections. Table 1 lists details of all antibodies used and their respective dilutions. For this protocol, a hematoxylin counterstain is not recommended.

### Quantification of trophoblast invasion

For quantification of trophoblast invasion, serial sections from 16 placentas were immuno-stained with an antibody against desmin and immuno-double-stained with antibodies against von Willebrand factor (vWF)/HLA-G. Within each section, only regions with trophoblast invasion were assessed for this study. A microscope (model DM6000B; Leica, Wetzlar, Germany) equipped with a motorized stage and a digital camera (model DP72; Olympus Austria GmbH, Vienna, Austria) was used for acquisition of 10 images per slide (magnification 200×). Within the serial sections, the same image section was selected. Images were obtained manually from 16 different placentas. To ensure objectivity, for 3 out of these 18 placentas the newCAST stereology software (Visiopharm, Horsholm, Denmark) was used for definition of a region of interest (ROI; regions with trophoblast invasion) and subsequent acquisition of ten systematically randomly selected images per slide within the ROI. The corresponding image section in the serial section with the desmin staining was taken manually again. For a proper overview, each image of vWF/HLA-G staining and the corresponding image from the serial section with the desmin staining were juxtaposed and converted together into a new image. All the resulting images were evaluated with the Zeiss AxioVision software version 4.8.2.0 (Carl Zeiss, GmbH, Vienna, Austria). In total, 320 images were analyzed quantitatively. In each image, every luminal cross section of a vessel was classified and counted by two independent observers as follows: artery invaded (A), artery with trophoblast attached (B), artery not invaded (C), vein invaded (D), vein with trophoblast attached (E), vein not invaded (F), vessel unclassified invaded (G), vessel unclassified with trophoblast attached (H), vessel unclassified not invaded (I). For quantification vessels which could not be classified due to negative desmin staining, size or other reasons, the category “vessel-unclassified” has been introduced besides the categories “artery” and “vein.” Data for this category have been excluded from this study since they do not add valuable information to the presented content. Detailed criteria for classification of vessels are listed in Table 2.

**Table 2:** Type of vessel-criteria for classification

Type of vessel	Definition
Artery–Arteriole	Positive staining for vWF, clear multi-layered (artery) or single-layered (arteriole) tunica media and/or positive staining for desmin
Vein–Venule	Positive staining for vWF, tunica media not visible, often collapsed, irregular shape, no staining for desmin
Vessel unclassified	Positive staining for vWF Vessels with diameter lower than ~10 µm (capillaries) Vessels completely surrounded by EVTs (maybe, the tunica media have already been completely replaced by EVTs) Vessels where a clear discrimination between artery and vein was not possible
*Properties*	*Definition*
Invaded	EVTs in the lumen of the vessel and/or endothelium replaced by EVTs
Attached	EVTs attached to the vessel wall from the outer side
Not invaded	EVTs in the surrounding interstitium, but not associated with the vessel

*EVTs* extravillous trophoblasts

### Controls

A subset of three out of the 16 placentas was assessed additionally with an immuno-staining against smooth muscle actin (smA) and hematoxylin and eosin (H&E) staining. The same subset was used for automated image acquisition by the VIS software and counted by one observer. Another subset of 10 out of the 16 placentas was assessed additionally with an immuno-staining against EphB4 and an immune-double staining against HLA-G and EphB4.

### Statistical analysis

Data are reported as means ± standard deviations. Student’s *t* test was applied for the quantification of EVTs replacing epi-/endothelium and EVTs in spatial proximity between glands and vessels, after testing for normal distribution (Kolmogorov–Smirnov test). Statistical analysis was done using SPSS IBM Statistics 21. A *p* value <0.05 was considered significant.

## Results

### Endovascular trophoblasts in arteries and veins: qualitative characteristics

**Fig. 1:**
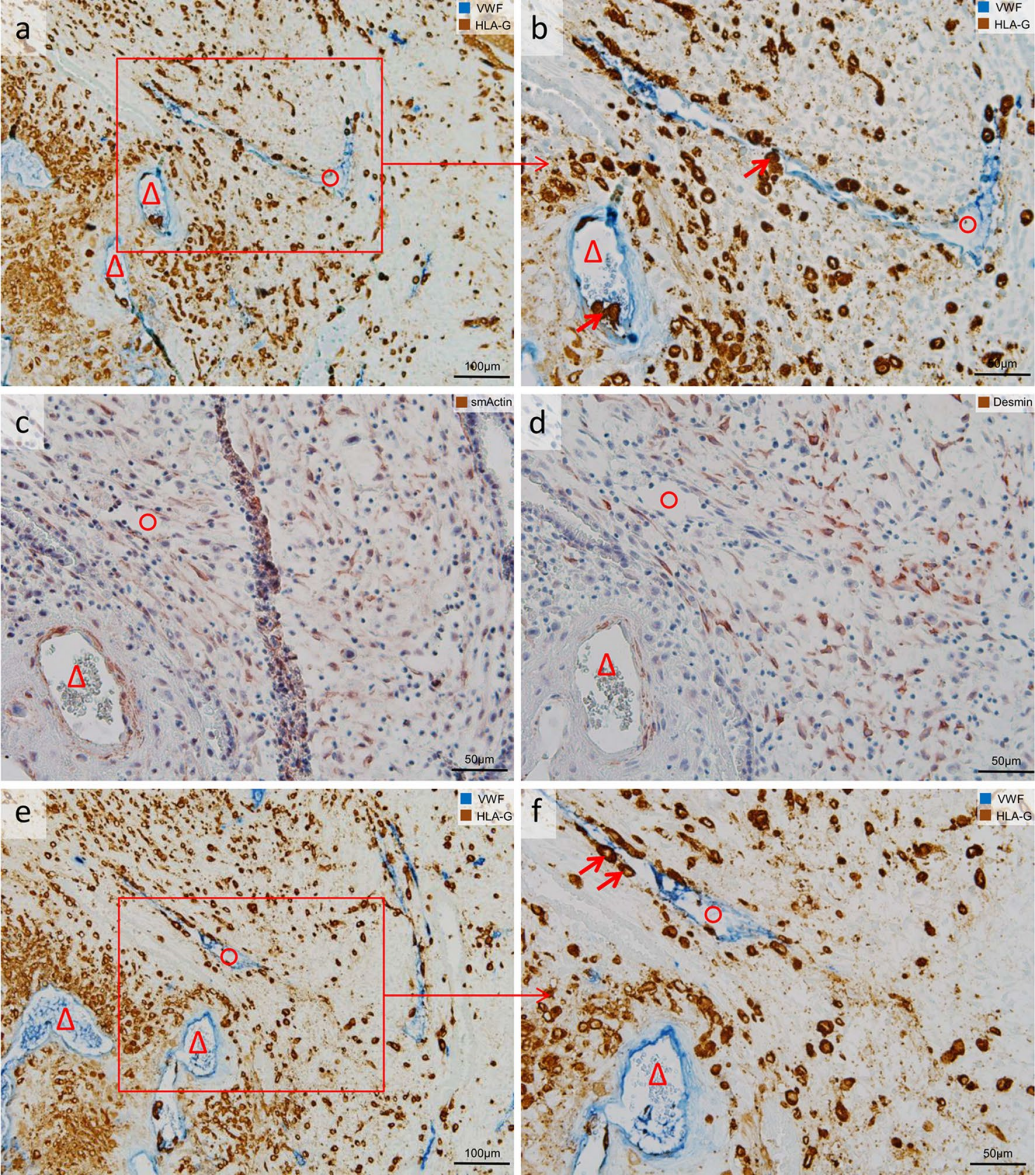
Uterine artery along with uterine vein invaded by extravillous trophoblasts (EVTs). Serial sections of invaded myometrium with immunohistochemical staining/double staining (gestational age 7 weeks). Images in **a**, **b**, **e**, **f** are immuno-double-stained for von Willebrand factor (VWF) (*blue* serves as marker for vascular endothelial cells) and major histocompatibility complex, class I, G (HLA-G) (*brown* serves as marker for EVT), **c** immuno-staining for smooth muscle actin (*brown* serves as marker for a muscle layer and as additional control for desmin staining; besides sm actin-positive myofibroblasts and smooth muscle cells of the myometrium stain positive), **d** immuno-staining for desmin (*brown* serves as marker for muscle layer). **a**, **e** Overview: Two sections through an invaded spiral artery (*triangle*) along with an invaded vein (*circle*). **b**, **f** Detail of the *inset* in **a** allows a closer look to the contour and shape of the invaded vessels. **c**, **d** Serial sections show the residual smooth muscle layer of the converted artery (*triangle*), but the complete absence of a muscle layer in the neighboring vein (*circle*). *Arrows* in **b**, **f** show single EVTs already situated in the lumen of the artery and vein. No nuclear counterstain (**a**, **b**, **e**, **f**) or nuclei were counterstained with hemalaun (**c**, **d**)

**Fig. 2:**
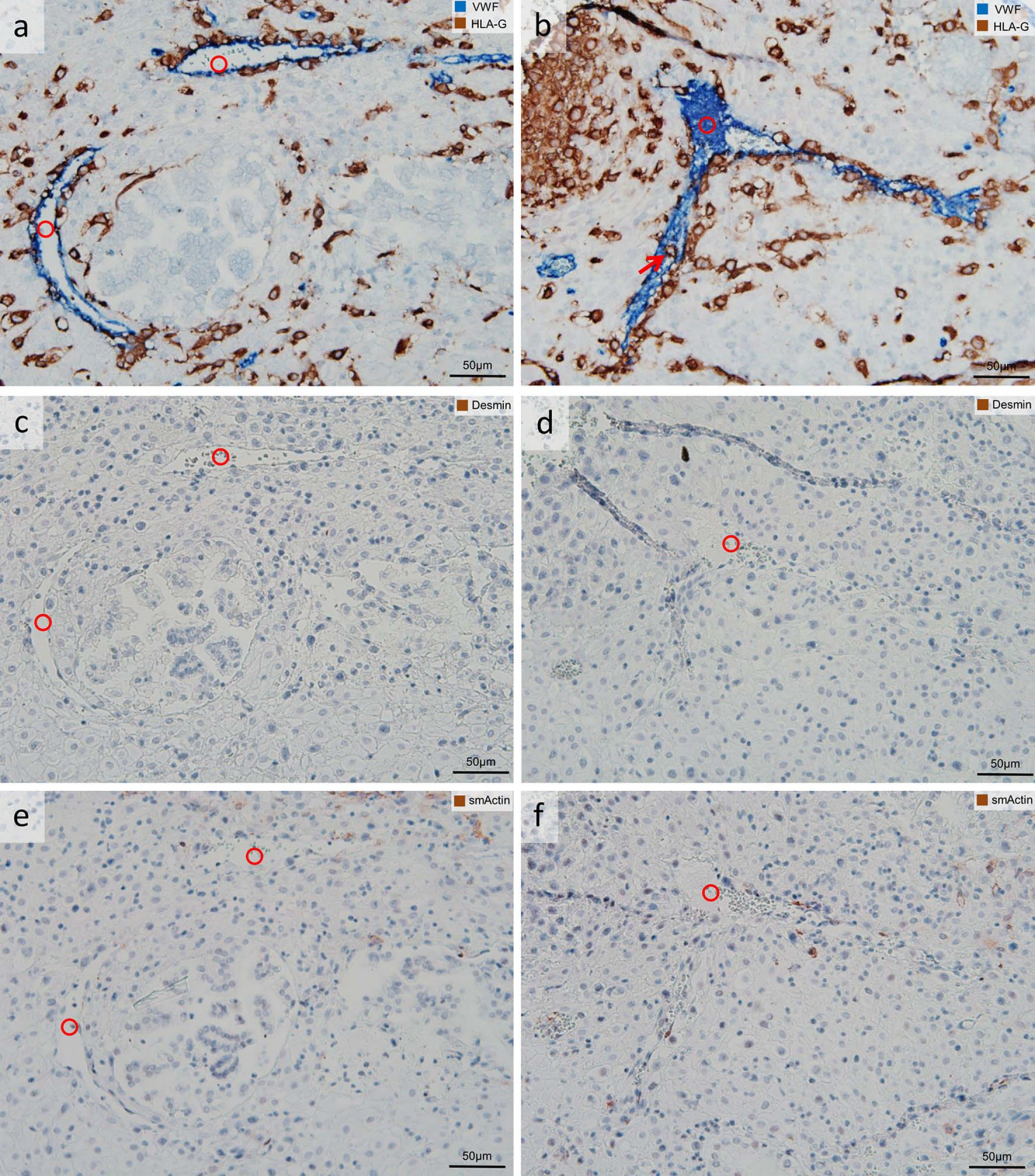
Uterine veins invaded by extravillous trophoblasts (EVTs). Columns are composed of serial sections of invaded decidua (gestational age 7 weeks). Sections are stained as follows: **a**, **b** immuno-double staining for von Willebrand factor (VWF) (*blue* serves as marker for vascular endothelial cells) and major histocompatibility complex, class I, G (HLA-G) (*brown* serves as marker for EVTs); **c**, **d** immuno-staining for desmin (*brown* serves as marker for a muscle layer); **e**, **f** immuno-staining for smooth muscle actin (*brown* serves as marker for a muscle layer and as additional control for desmin staining; besides some sm actin-positive myofibroblasts are seen). **a**, **b** Uterine vessels (*blue*), the decidual stroma is invaded by EVTs (*brown*), and the endothelium of the vessel is partly replaced by EVTs (**a**), whereas the *arrow* in **b** points to a single EVT situated in the lumen of the vein. **c**–**f** The absence of a muscular layer confirms that the vessels in **a**, **b** are uterine veins (*circle*). No nuclear counterstain (**a**, **b**) or nuclei were counterstained with hemalaun (**c**–**f**). *Circles* uterine veins

Endovascular trophoblasts were visualized with the specific immuno-double staining against vWF/HLA-G in sections of invaded first trimester placental decidua basalis. Serial sections were immuno-stained with antibodies against desmin. For a proper overview, each image of vWF/HLA-G staining and the corresponding image from the serial section with the desmin immuno-staining were juxtaposed (Figs. 1b, d, 2a–d, 4a, b, 5a–f) and converted together into a new image. This enables the classification between vessel invaded/trophoblast attached/not-invaded and in parallel the discrimination between artery and vein. Identification of arteries and veins was primarily based on vascular morphology in H&E stained decidua sections according to the following criteria (also listed in Table 2). An artery was characterized by a squamous endothelium, and a thick tunica media composed of several layers of smooth muscle cells and fibroblasts. Due to the large amount of smooth muscle cells, the lumen appears round-shaped in a histological cross section. A vein/venule was lined by an endothelium, but with a thinner tunica media including only a very weak or no muscular layer. Veins are mostly collapsed in histological FFPE specimens; therefore, the profile in histological cross sections does not appear round/oval but rather irregular. Beside these basic morphological characteristics, immuno-staining of the muscular layer with antibodies against desmin and smooth muscle actin (smA) strongly supports the discrimination between arteries and veins.

**Fig. 3:**
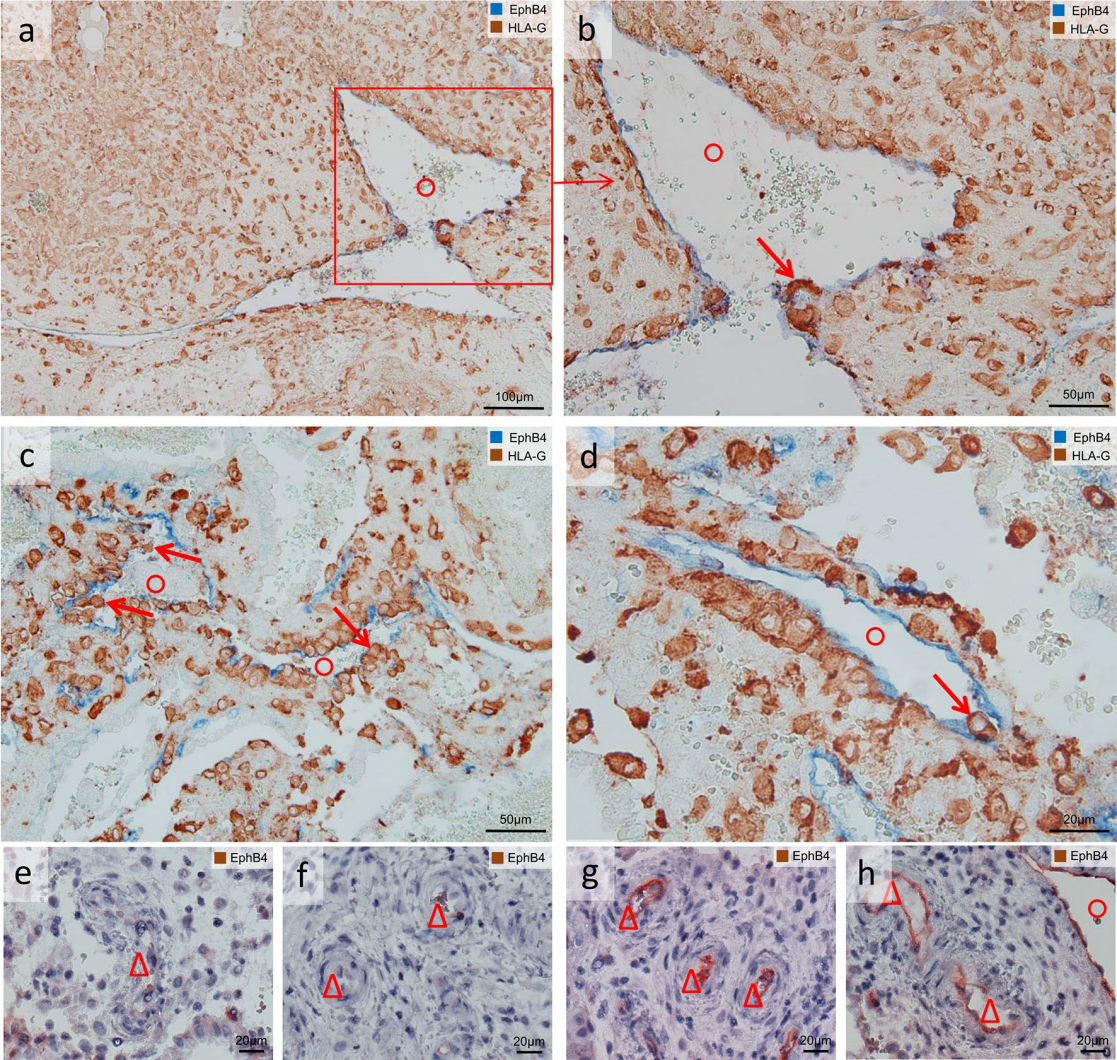
Extravillous trophoblasts (EVTs), uterine veins and EphB4. Sections of invaded decidua with immunohistochemical staining/double staining (gestational age 7–8 weeks). Images in **a**–**d** are immuno-double-stained for EphB4 (*blue* serves as marker for venous endothelial cells) and major histocompatibility complex, class I, G (HLA-G) (*brown* serves as marker for EVT), sections in **e**–**h** are immuno-stained for EphB4. **a** Overview: Eph4-positive endothelium (*blue*) of a vein (*circle*); the surrounding decidual stroma is invaded by EVTs (*brown*). **b** Detail of the *inset* in **a** allows a closer look to the invaded vein (*circle*), *arrow* points to EVT within the endothelium. *Arrows* in **c**, **d** indicate additional EVTs within the venous lumen and/or the venous endothelium. **e**–**h** Morphologically unambiguous arteries (*triangles*) usually stain negative for EphB4 (**e**, **f**), but occasionally the arterial endothelium displays positive staining for EphB4 (**g**, **h**). No nuclear counterstain (**a**–**d**) or nuclei were counterstained with hemalaun (**e**–**h**). *Circles* uterine veins. *Triangles* uterine arteries

**Fig. 4:**
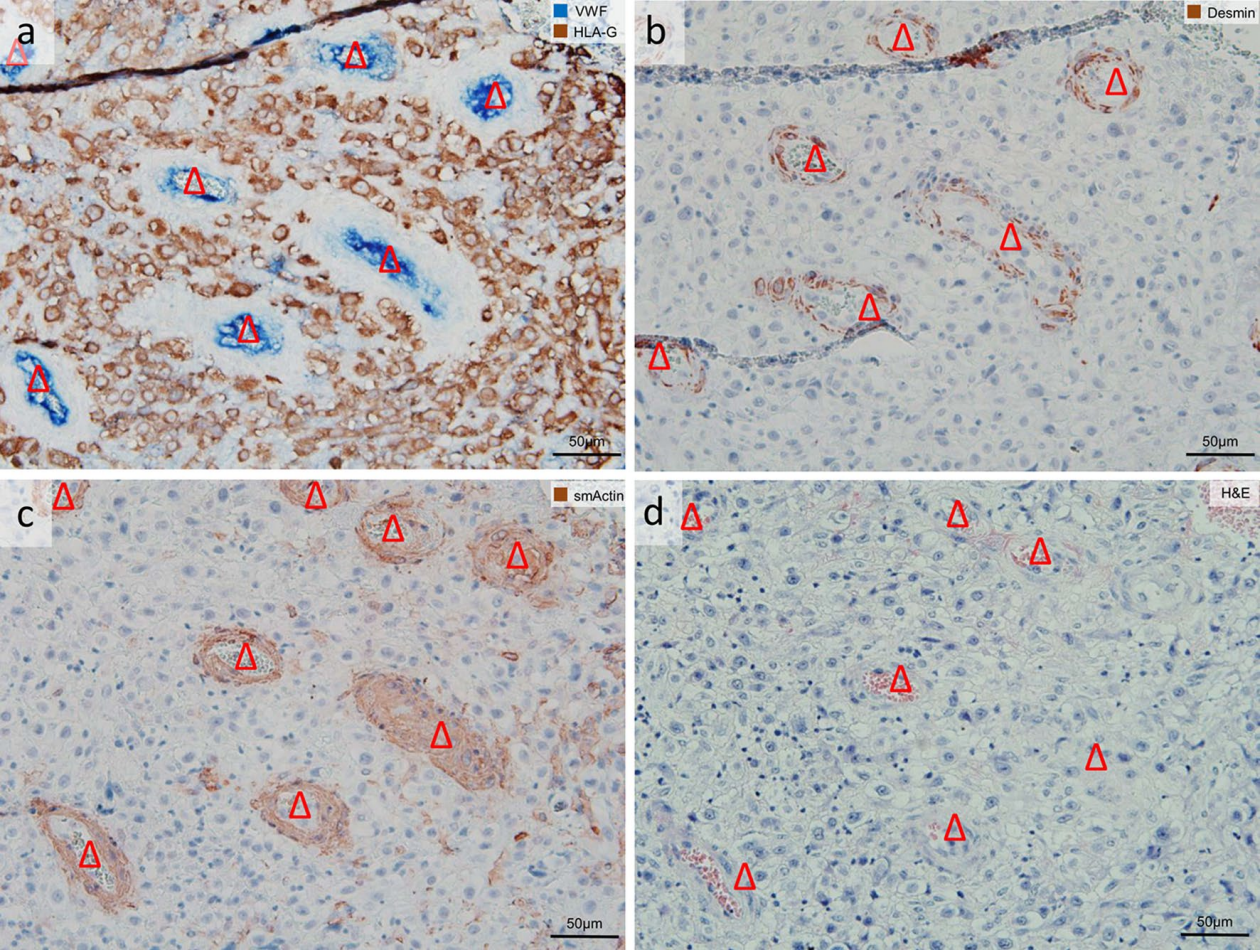
Uterine artery not invaded by extravillous trophoblasts (EVTs). Serial sections of invaded decidua (gestational age 7 weeks). Images are stained as follows: **a** immuno-double staining for von Willebrand factor (VWF) (*blue* serves as marker for vascular endothelial cells) and major histocompatibility complex, class I, G (HLA-G) (*brown* serves as marker for EVTs), **b** immuno-staining for desmin (*brown* serves as marker for a muscle layer), **c** immuno-staining for smooth muscle actin (*brown* serves as marker for a muscle layer and as additional control for desmin staining, besides some sm actin-positive myofibroblasts are seen), **d** hematoxylin and eosin staining. **a** Uterine vessels (*blue* *triangles*); the decidual stroma is strongly invaded by EVTs (*brown*). **b**–**d** The presence of a muscular layer confirms that the vessel is a uterine artery (*triangle*). No nuclear counterstain (**a**) or nuclei were counterstained with hemalaun (**b**–**d**). *Triangles* uterine artery

**Fig. 5:**
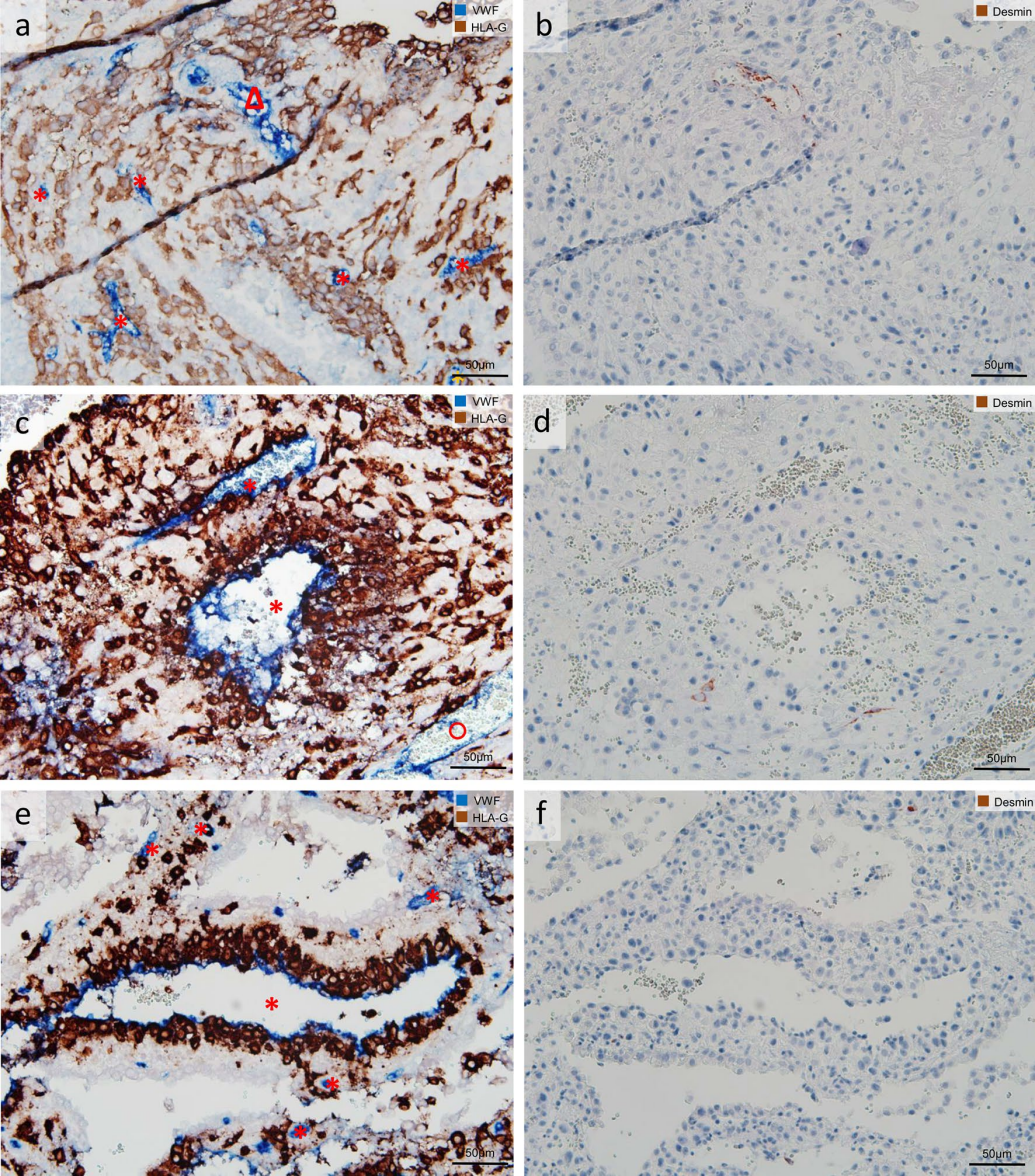
Examples for unclassified vessels (*asterisk*). Invaded decidua with immuno-staining and double staining (gestational age 7–8 weeks). *Rows* are composed of serial sections **a**–**b**, **c**–**d**, **e**–**f**, and sections in the *left column* are immuno-double-stained for von Willebrand factor (vWF) (*blue* serves as marker for vascular endothelial cells) and major histocompatibility complex, class I, G (HLA-G) [*brown* serves as marker for extravillous trophoblasts (EVTs)], in the *right column* immuno-stained for desmin (*brown* serves as marker for muscle layer). *Asterisks* mark unclassified vessels, *triangle* marks an artery, and* circles* mark veins. **a**, **b** Examples for unclassified vessels with a diameter below 10 µm. **c**–**f** Examples for unclassified vessels completely surrounded by EVTs; a possible layer of smooth muscle cells may have already been replaced by EVTs. No nuclear counterstain (**a**, **c**, **e**) or nuclei were counterstained with hemalaun (**b**, **d**, **f**)

EVTs were observed in the decidual stroma (Figs. 1, 2, 3, 4, 5, 7), associated with uterine arteries (Fig. 1), but also with uterine veins (Figs. 1, 2, 3). Moreover, single EVTs were also observed in the lumen of arteries (Fig. 1b arrow) and in the lumen in veins (Figs. 1b, 2b, 3b–d arrows). We repeatedly observed arteries in strongly invaded regions of the decidua that were not (yet) invaded by EVTs (Fig. 4). Assessment of serial sections showed that the same artery may be invaded by EVTs in other proportions of the vessel (data not shown).

In one-third of the cases, despite the application of the above-described morphological criteria and the desmin staining in the serial section, a classification between arterial or venous vessels was not possible. This applied mostly for vessels with a diameter smaller than ~10 µm (Fig. 5a–b) and for vessels completely surrounded by EVTs (Fig. 5c–f). For the latter, it could be speculated that the tunica media have already been completely replaced by EVTs. For the quantification approach, all these vessels were defined as “vessels unclassified” and were excluded from semi-quantitative analysis (33 % of all counted vessels). The detailed criteria for inclusion and exclusion to the arterial/venous system are listed in Table 2.

### Endovascular trophoblasts in arteries and veins: quantitative analysis

**Fig. 6:**
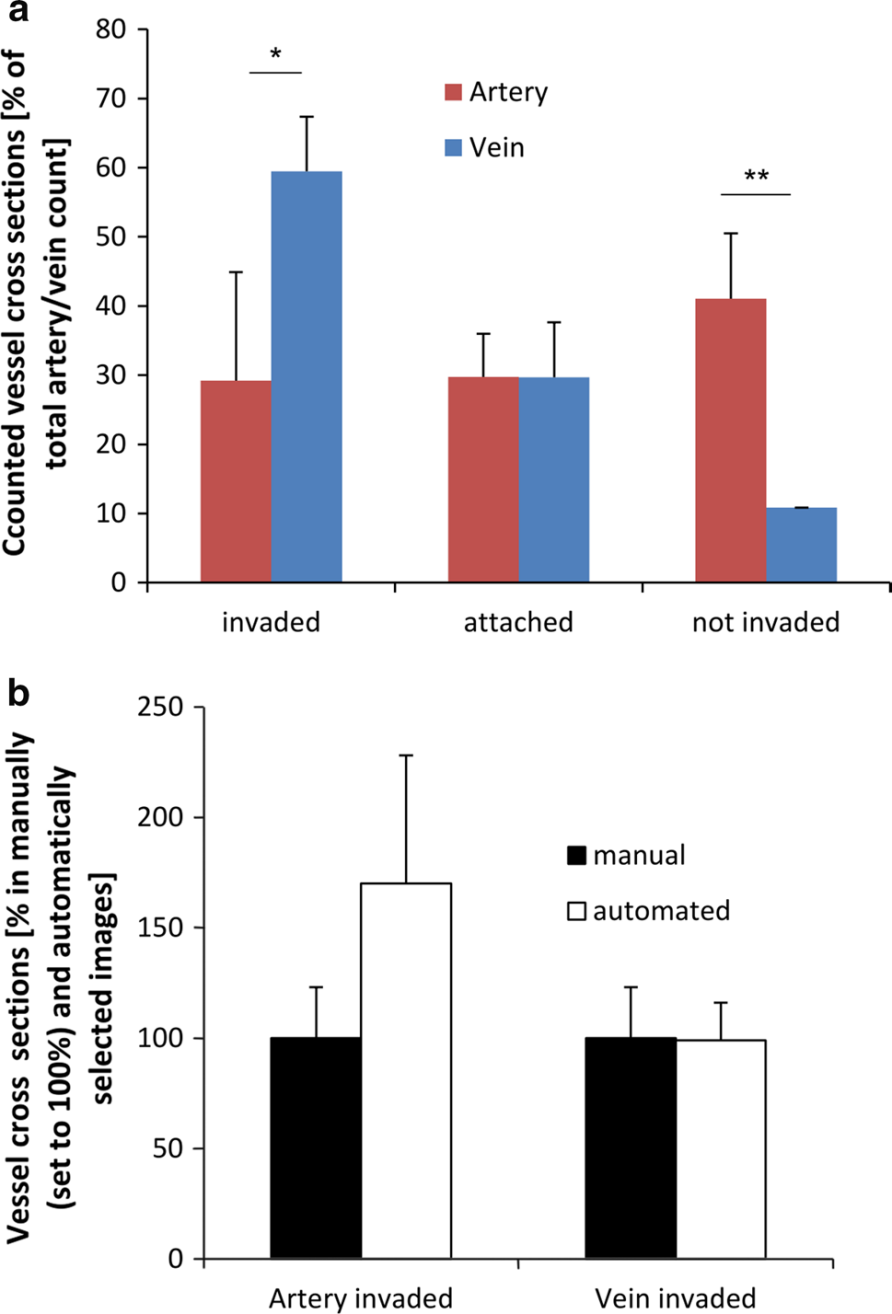
Quantification of trophoblast invasion: Figure shows that the number of invaded venous cross sections is higher than the number of invaded arterial cross sections. **a** Counted vessel cross sections of 16 placentas revealed significantly higher EVT invasion into veins compared to arteries (two observers). EVTs attached to the respective vessel showed similar results with no significant differences in numbers. In all assessed specimens, significantly more uninvaded arteries were found compared to uninvaded veins. **b** The comparison between manual and automated image selection (one observer) did not reveal any significant differences within the subset of three placentas, neither in invaded arteries, nor in invaded veins. Manual counting was set to 100 %. *p < 0.05; **p < 0.01

Counted vessel cross sections revealed significantly higher EVT invasion into veins (59.5 ± 7.9 %) compared to arteries (29.2 ± 15.7 %) (Fig. 6a). EVTs attached to the respective vessel type showed similar results with no significant difference in numbers (arteries: 29.8 ± 6.2 %; veins: 29.7 ± 7.9 %) (Fig. 6a). In all assessed specimens, significantly more uninvaded arteries were found (41 ± 9.5 %) compared to uninvaded veins (10.8 ± 0.01 %) (Fig. 6a).

### Control 1: Comparison—manually and automated image acquisition

In a subset of three placentas, results revealed no significant differences between the two image selection methods (manually or automated systematically randomly selected). Setting the value for manual selection to 100 %, neither in invaded arteries (manual: 100 ± 23 %; automated: 170 ± 58 %), nor in invaded veins (manual: 100 ± 23 %; automated: 99 ± 17 %) a significant difference could be detected (Fig. 6b).

### Control 2: Additional smA and H&E staining

As a control for the setup of quantification described above, in a subset of three placentas respective serial sections were additionally immuno-stained with an antibody against smooth muscle actin (smA). The immuno-staining against smA confirmed no or only a weak muscular layer in the vessels classified as vein (Fig. 2e, f). Serial sections from the same subset were additionally stained with hematoxylin and eosin (H&E) for a clear and precise morphology (Fig. 4d). Both smA-immuno-staining and H&E-staining confirmed the classification into artery and vein, previously performed with combination vWF/HLA-G double staining and desmin staining.

Discrimination between arteries and veins was additionally tested with immune-single and immune-double staining with the venous marker EphB4. Several Eph4-positive vessels were associated with and invaded by EVTs (Fig. 3a–d). Morphologically unambiguous arteries react usually negative with EphB4 (Fig. 3e, f), but sporadically the arterial endothelium displays positive staining for EphB4 (Fig. 3g, h).

### Glandular epithelial cells in the lumen of vessels

**Fig. 7:**
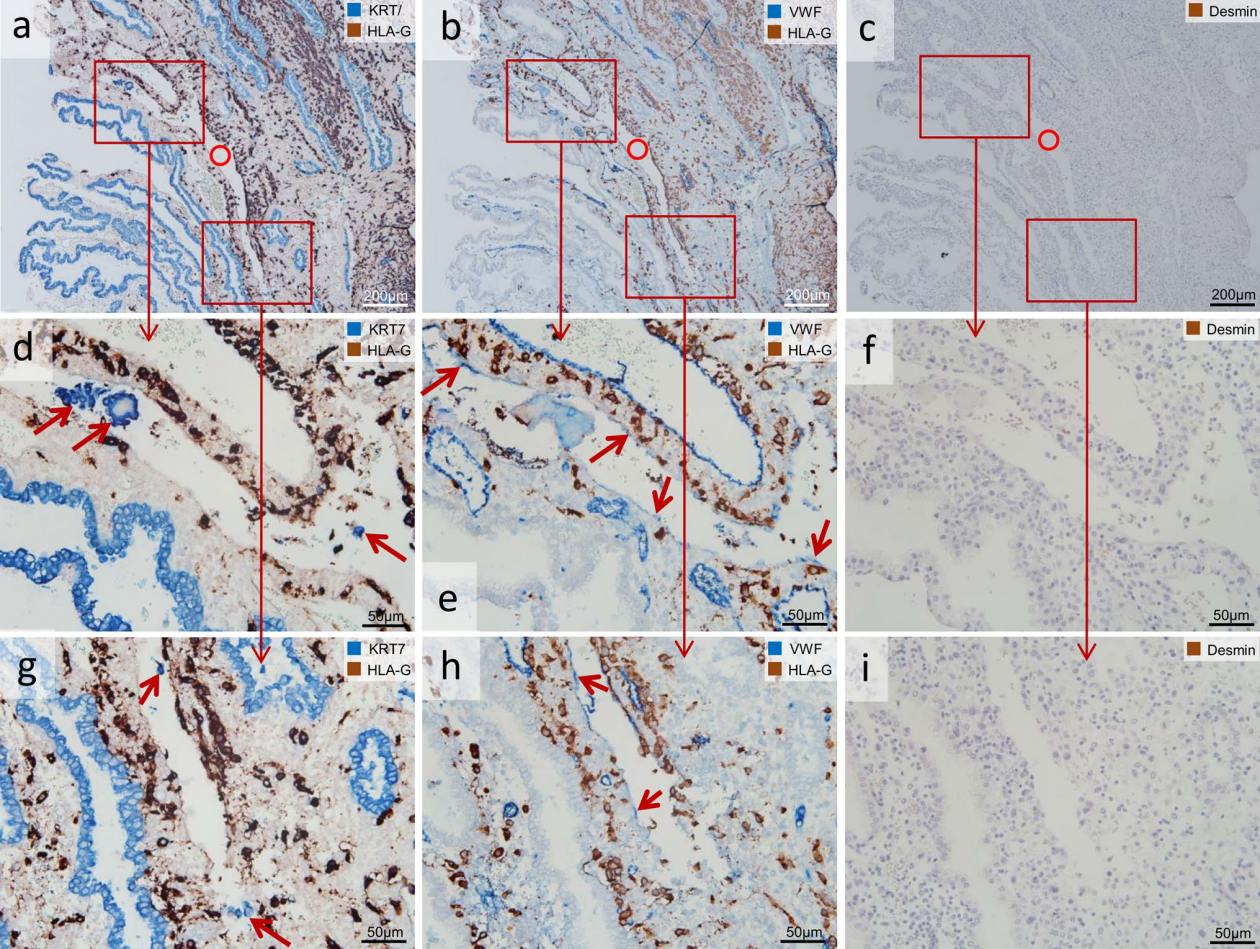
Glandular epithelial cells and syncytial fragments in uterine veins. Serial sections of invaded decidua (gestational age 8 weeks). Sections in the *left column* (**a**, **d**, **g**) are immuno-double-stained for keratin 7 (KRT7) (*blue* serves as marker for glandular epithelial cells and syncytial fragments) and major histocompatibility complex, class I, G (HLA-G) (appears *dark brown* serves as marker for extravillous trophoblast), in the *middle column* (**b**, **e**, **h**) von Willebrand factor (vWF) (*blue*, serves as marker for vascular endothelial cells) and major histocompatibility complex, class I, G (HLA-G) (*brown*), in the *right column* (**c**, **f**, **i**) immuno-staining for desmin (*brown* serves as marker for muscle layer). (**a**, **d**, **g**) In the lumen of the vein (*circle*) detached glandular epithelial cells (**d**, **g**) and putative syncytial fragments can be found (**d**) (*arrows*, KRT7-positive (*blue*), but HLA-G-negative). **b**, **e**, **h** The serial sections in the *middle* and *right column* show that this luminal structure is a vessel (*circle* in **b**, **c**), *arrows* in **e**, **h** show the endothelium (vWF-positive), and the endothelium is partly replaced by EVTs. **c**, **f**, **i** The *right column* confirms the venous origin of the vessel (*circle*) by negative staining for desmin. The endothelium in this vein is incomplete. No nuclear counterstain (*left* and *middle column*) or nuclei were counterstained with hemalaun (*right column*)

In the lumen of some veins, also KRT7-positive (appearing blue in the immunohistochemical double staining), but HLA-G-negative cells could be observed (Fig. 7d, g). This staining pattern is characteristic for (detached) glandular epithelial cells and syncytial fragments.

## Discussion

“Uterine arteries are invaded by extravillous trophoblast, while veins are not” is the generally accepted opinion in placental research and teaching. Maybe, we need to expand our view! Our data show that uterine veins are invaded by EVTs even to a greater extent than uterine arteries in early pregnancy. Besides that, we found detached glandular epithelial cells or syncytial fragments (KRT7-positive, but HLA-G negative) in the lumen of such veins. Structural identification is considerably facilitated by application of respective immunohistochemical markers. As previously suggested, we recommend the utilization of immunohistochemical double staining for HLA-G in combination with vWF/KRT7 as specific markers for extravillous trophoblasts to avoid potential misinterpretation between EVTs and glandular epithelial cells (Moser et al. 2011).

The number of counted vessel cross sections varied considerable between the individual placentas. However, invaded arteries and veins were found in all assessed placentas. Notably, more than every second venous cross section was invaded by EVTs. Vessels completely surrounded by EVTs have been excluded from quantification, since it could be speculated that here the tunica media of an artery have already been completely replaced by EVTs. Approximately one quarter of the excluded “vessels unclassified” were vessels completely surrounded by EVTs. For this quarter, it could be speculated that all of them were completely remodeled arteries. However, even if these “arteries” would be added to the arterial invasion, there is still an approximately equal invasion toward arteries and veins. This exclusion may skew our results and needs to be mentioned as a possible limitation.

So far, the focus was on the invasion and transformation of uterine spiral arteries and it seems as if invasion of veins has been ignored to date. In Benirschke et al. (2006) uteroplacental veins of the first trimester are described as “Endothelial tubes, surrounded by few regressive medial and adventitial cells, embedded in decidua and extravillous trophoblast cells; the latter rarely invade the venous walls and never the venous lumina” (Benirschke et al. 2006). However, we repeatedly observed EVTs in the lumen of veins, and Fig. 2b provides respective evidence. Also Pijnenborg et al. (2006) stated it as fact that endovascular invasion only occurs within arteries and never into veins, but the authors did not provide any evidence for this (Pijnenborg et al. 2006).

A look into the historical literature shows that already in 1941 decidual capillaries and venules were observed to link with the trophoblast lacunae at 16 days of development (Hertig and Rock 1941; Schneider and Moser 2016). Decades later, Kam et al. (1999) described that the veins in the uterine mucosa in the absence of trophoblast showed no endothelial swelling and few actin-positive medial cells, whereas in the decidua basalis trophoblasts were present around some veins, but without modification of vessel walls (Kam et al. 1999). Craven et al. (2000) investigated tissues from 100 first trimester placentas; all of these decidual tissues had dilated veins. They described that extravillous trophoblast cells were attached to the endothelium and cells appeared to invade the underlying stroma. Moreover, they described the presence of placental villi and cell islands (described as clusters of mononuclear trophoblast cells) in the lumen of uterine veins in the decidua and deep within the myometrium. These cell islands were completely detached from the placental villi, confirmed by serial sections (Craven et al. 2000).

It would be of great interest to stain such serial sections with the respective markers to distinguish between fetal villous and extravillous trophoblast cells and maternal glandular epithelial cells. We suggest that also detached uterine glandular epithelial cells are passing uteroplacental veins and thus can be found in the lumen of such veins. These epithelial cells have been removed from their original site, uterine glands, by invading endoglandular trophoblasts (Moser et al. 2015), have passed the intervillous space and are now drained into the maternal circulation.

In their study from 2000, Craven et al. suggest that trophoblast cell islands and villi enter maternal veins, floating villi implant in veins (as part of lateral placental growth), and link this observation with the transformation of the venous wall to a new basal plate tissue (Craven et al. 2000). The same authors compared and examined in another study the late secretory endometrial biopsy with first trimester decidua (Craven and Ward 1999) and showed that the decidual veins were dilated, but there were no dilated veins in the secretory endometrium. The dilated veins in the decidua often contained syncytiotrophoblastic fragments; many of them associated with blood clots. There was no correlation of the gestational age of a case with the number of syncytiotrophoblast fragments in the uteroplacental veins (range of gestational age 7–11 weeks) (Craven and Ward 1999). In another study, Craven et al. (2002) identified fibrin deposition in decidual veins that was associated with trophoblast cell invasion. They also described that the endothelium became incomplete at sites of trophoblast attachment; this was accompanied with the deposition of fibrin along the vein wall (Craven et al. 2002). We also observed an incomplete endothelium in veins (Fig. 7). However, in all of their studies Craven et al. were indeed describing attachment of trophoblast cells from the luminal side, but they never described invasion into veins (Craven and Ward 1999; Craven et al. 2000, 2002). Besides that, we want to suggest that the fibrin described by Craven et al. 2002 represents actually fibrin-type fibrinoid according to the definition in (Kaufmann et al. 1996).

We frequently observed non-invaded arteries in invaded and also strongly invaded decidual regions (Fig. 4). Despite massive EVT invasion in the surrounding, there appears no loss of the smooth muscle layer in these arteries. This is a quite common occurrence, but—to the best of our knowledge—has not been mentioned yet in the literature. Assessment of serial sections showed that the same artery may be invaded by EVTs in other portions of the vessel (data not shown).

**Fig. 8:**
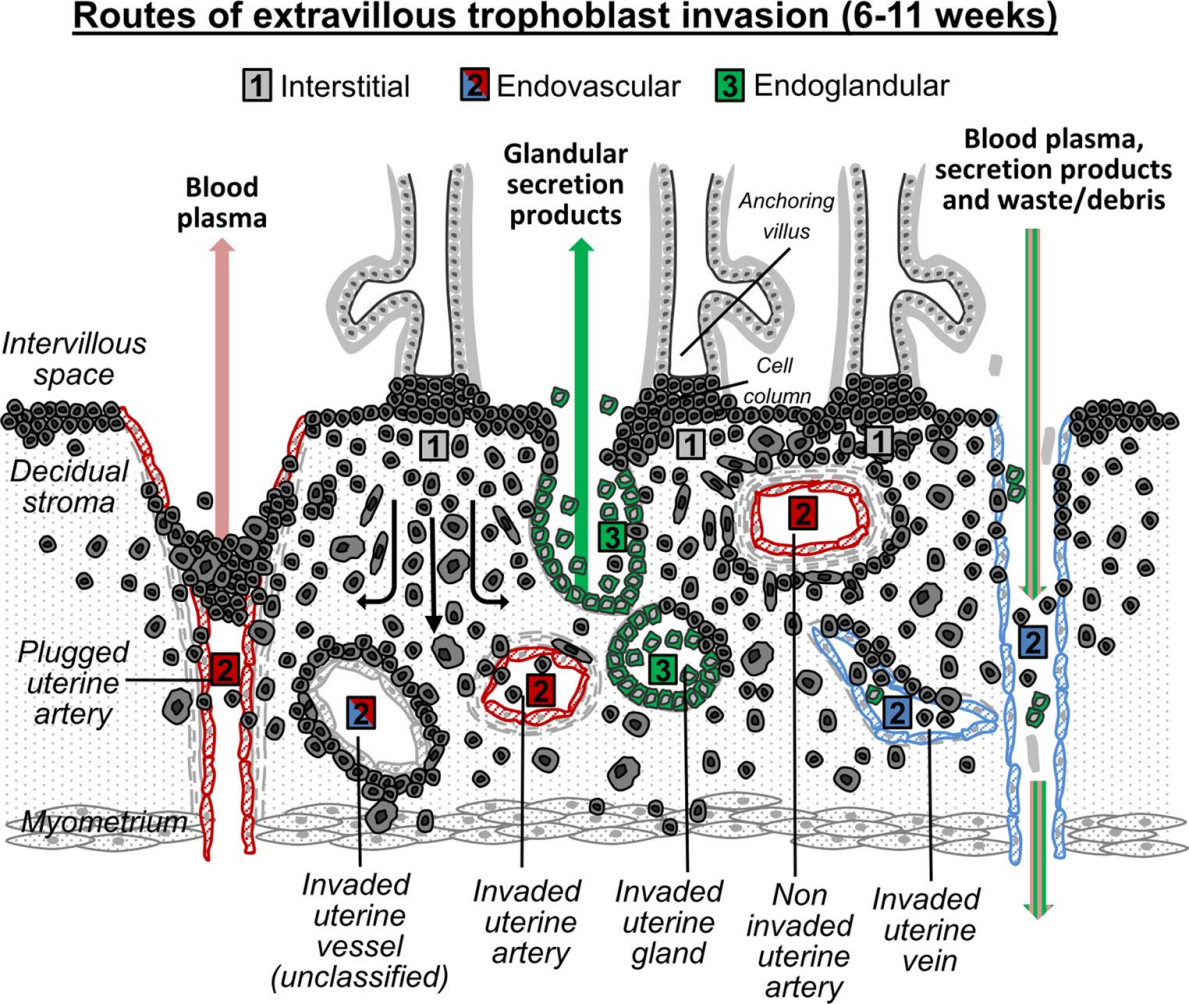
Concept of extravillous trophoblast (EVTs) invasion in first trimester human placenta. EVTs originate from cell columns of anchoring villi. During the first trimester of pregnancy, EVTs invade into the decidual interstitium (*1*) reaching the inner third of the myometrium, thereby anchoring the placenta to the uterus. They also follow the (*2*) endovascular route of invasion, plug, line and remodel spiral arteries (*2*, *red*), thus being responsible for the establishment of the maternal–fetal blood flow starting with the beginning of the second trimester. Prior to the opening of spiral arteries toward the intervillous space, maternal blood plasma is seeping through the trophoblastic plugs. Endovascular trophoblasts also reach and invade uterine veins and replace the venous endothelium (*2*, *blue*). Via the opened and dilated veins, maternal blood plasma and glandular secretion products are drained from the intervillous space into the maternal circulation. Endovascular trophoblasts invade and replace the tunica media of vessels, which in some cases leads to the fact that classification into artery or vein is no longer possible (*2*, *red* and *blue*). Endoglandular trophoblasts (*3*) are situated nearby uterine glands, replace the glandular epithelium and open the lumen of uterine glands toward the intervillous space. Scheme adapted from (Moser et al. 2015)

EVT invasion into uterine veins is not a rare event (Fig. 6). Invasion into uterine veins occurred in all placentas assessed throughout the first trimester (time window: 6–11 weeks of gestation). No correlation with gestational age could be detected; maybe, the sample size was too small to address this aspect. Also Smith et al. (2009) did not see any association between arterial remodeling and gestational age (Smith et al. 2009). Only few large trophoblastic plugs were identified in the assessed specimens, sporadic in some spiral arteries, never in the invaded veins. Rarely, aggregations of few EVTs (<5 cells/vessel cross section) were detected in the venous lumina. This is in contrast to the directed invasion, remodeling and plugging of spiral arteries by endovascular trophoblasts. On the one hand, due to this large amount of venous trophoblast invasion during the first trimester, it is tempting to speculate that venous EVT invasion is—similar to the invasion of spiral arteries—a directed and controlled process and responsible for the early opening of uterine veins toward the intervillous space for a rapid draining of blood plasma, glandular secretion products and cellular waste from the intervillous space (Fig. 8). The cellular waste may also include fragments scaled off from trophoblast plugs of spiral arteries, which have reached the venous lumen via the intervillous space. On the other hand, we propose—and we are aware that this is an audacious thesis—that none of the routes of extravillous trophoblast invasion (interstitial, endovascular, endoglandular) is specifically directed, suggesting that EVTs are just highly invasive cells and simply attach to and invade into each and every structure which gets into the way of invading trophoblasts. Evidence for this thesis is provided by the fact that in vitro EVTs attach and invade to various offered matrices like collagen I (Lacey et al. 2002), laminin (Seeho et al. 2008) and Matrigel (Genbacev et al. 1993) beside decidual tissue. There is also respective evidence from in vivo scenarios, since embryos attach to and invade into the tissues of fallopian tubes, ovaries and the peritoneum.

There is still only limited knowledge on the specific types of fetal cells circulating in maternal blood. Recently, fetal cells in maternal blood were found to express a typically extravillous gene pattern. Due to this gene expression pattern, the authors suggest that it is likely that a part of the fetal cells present in maternal blood are EVTs. These cells exhibit expression of markers with invasive capability, for endothelial cell-to-cell adhesion and at the same time retaining epithelial markers (Hatt et al. 2014). We suggest that these EVTs reach the maternal circulation via invasion of uterine arteries and veins as well as via the endoglandular pathway: EVTs invade uterine glands and are flushed together with the glandular secretion products (and detached glandular epithelial cells) toward the intervillous space. Putative syncytial fragments, KRT7-positive, but HLA-G negative, may be present in the lumen of invaded veins as well. Finally, they are drained into the maternal circulation via the opened and already dilated uterine veins. This thesis may also be an explanation why uterine veins are dilated in the decidua during very early pregnancy.

According to Smith et al. (2009) there are trophoblast-independent and trophoblast-dependent phases of remodeling; extensive disruption and remodeling of the spiral arteries occur before endovascular colonization by EVTs and are coincident with vascular infiltration by uNK and macrophages (Smith et al. 2009). Taken together, even with visualization of the smooth muscle layer, and since the majority of smooth muscle cell loss from the spiral arteries in early stages of remodeling occurs before EVT presence (Smith et al. 2009), the discrimination between a vein with little smooth muscle and a remodeled artery that has lost all its smooth muscle cells is tricky. Ephrin-B2, an Eph family transmembrane ligand, and its receptor EphB4 have been attributed to be markers for arteries and veins, respectively (Wang et al. 1998). These ligand–receptor interactions with various members of the ephrin/eph family may stimulate cytotrophoblast migration toward arteries and veins (Red-Horse et al. 2005). Another study reported a more widespread pattern of ephrin/eph expression throughout the vascular system (Adams et al. 1999). It needs to be mentioned that the studies by Wang et al. (1998) and Adams et al. (1999) have been conducted with mice and not with human specimens. Human placental venous endothelial cells did not overexpress EphB4 compared to arterial endothelial cells as demonstrated by Lang et al. (2008). We observed that EphB4 occasionally stains the endothelium of morphologically unambiguous arteries (Fig. 3g, h). Thus, EphB4 may serve as helpful marker and additional control, but one should refrain from taking it as a single criterion for discrimination between arteries and veins.

However, and viewed from the opposite side, the majority of all vWF-positive vessels in the invaded decidua basalis are associated with and invaded by EVTs. From this point of view, it seems questionable whether it can be true that the majority of all vessels are arteries and why the venous part should be so extremely underrepresented in the decidua? And even if some or even numerous of the vessels have been classified wrongly, the fact that the majority of the vessels are invaded by EVTs gives enough hint for a venous invasion by EVTs.

In conclusion, EVTs strongly invade uterine veins beside the decidual stroma, spiral arteries and uterine glands. Detached glandular epithelial cells or putative syncytial fragments (KRT7-positive, but HLA-G negative) can be detected in the lumen of such veins. All potential routes of EVTs invasion during the first trimester are schematically represented in Fig. 8. Because of the large amount of venous EVT invasion, we suggest that there is a biological relevance to invade and open veins toward the intervillous space for draining of blood plasma, glandular secretion products and debris before the establishment of the uteroplacental blood flow at the end of the first trimester. Whether or not pathologies derive from failures of venous invasion has not been addressed yet but may be the subject of further investigation.
